# Chromosomal inversions from an initial ecotypic divergence drive a gradual repeated radiation of Galápagos beetles

**DOI:** 10.1126/sciadv.adk7906

**Published:** 2024-05-31

**Authors:** Carl Vangestel, Janne Swaegers, Zoë De Corte, Wouter Dekoninck, Karim Gharbi, Rosemary Gillespie, Matthias Vandekerckhove, Steven M. Van Belleghem, Frederik Hendrickx

**Affiliations:** ^1^ Royal Belgian Institute of Natural Sciences, Brussels, Belgium.; ^2^ Terrestrial Ecology Unit, Biology Department, Ghent University, Gent, Belgium.; ^3^ Ecology, Evolution and Conservation Biology, Biology Department, University of Leuven, Leuven, Belgium.; ^4^ Earlham Institute, Norwich Research Park, Norfolk, United Kingdom.; ^5^ Department of Environmental Science, Policy, and Management, University of California, Berkeley, Berkeley, CA, USA.

## Abstract

Island faunas exhibit some of the most iconic examples where similar forms repeatedly evolve within different islands. Yet, whether these deterministic evolutionary trajectories within islands are driven by an initial, singular divergence and the subsequent exchange of individuals and adaptive genetic variation between islands remains unclear. Here, we study a gradual, repeated evolution of low-dispersive highland ecotypes from a dispersive lowland ecotype of *Calosoma* beetles along the island progression of the Galápagos. We show that repeated highland adaptation involved selection on multiple shared alleles within extensive chromosomal inversions that originated from an initial adaptation event on the oldest island. These highland inversions first spread through dispersal of highland individuals. Subsequent admixture with the lowland ecotype resulted in polymorphic dispersive populations from which the highland populations evolved on the youngest islands. Our findings emphasize the significance of an ancient divergence in driving repeated evolution and highlight how a mixed contribution of inter-island colonization and within-island evolution can shape parallel species communities.

## Teaser

How the spread of an ancient divergence blends the role of colonization and evolution in a repeated island radiation.

## INTRODUCTION

Insular radiations, like those found on island archipelagos, provide natural laboratories to study the ecological and evolutionary drivers of adaptation and species diversification (*1*–*3*). In particular, when similar forms evolved repeatedly within separate islands, as observed for, e.g., Caribbean *Anolis* lizards (*4*, *5*), Hawaiian spiders (*6*, *7*), and *Oreinotinus* plants in the Central and South American cloud forest archipelago (*8*), these replicated radiations have demonstrated that the direction of evolution can be unexpectedly predictable and that niches within an island can be filled either by species that colonized the island or through in situ radiations (*2*, *9*, *10*). However, the extent to which these repeated radiations within islands represent independent evolutionary replicates remains poorly understood (*11*–*14*).

**Fig. 1.**
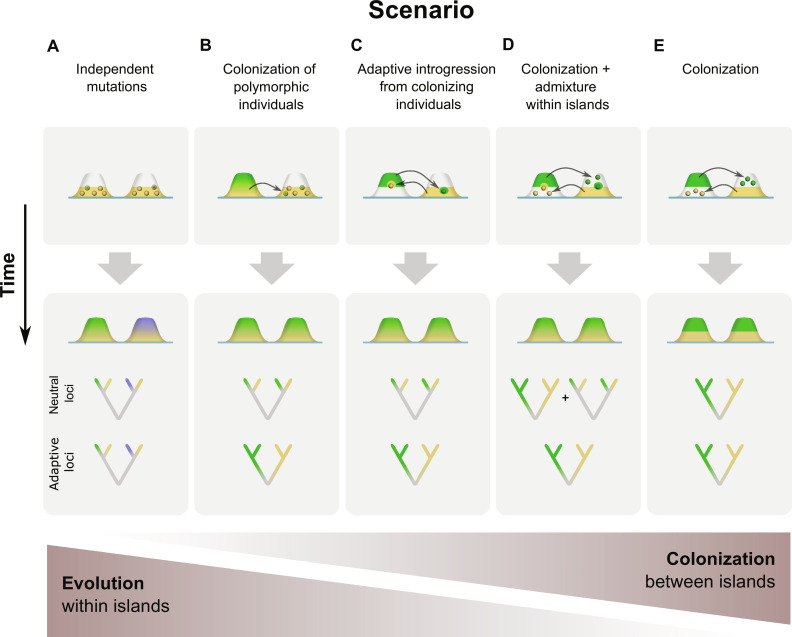
Proposed scenarios leading to parallel species pairs on two hypothetical islands and how this is driven by the contribution of evolution within islands versus colonization between islands. Island areas in yellow are occupied by a lowland ecotype, areas in green or blue are occupied by a highland ecotype, and white areas represent unoccupied habitat. Blended colors represent admixture between both ecotypes. The top panel depicts the onset of the evolutionary or colonization process. The bottom panel depicts the outcome of species pair formation and expected phylogenetic signals. (**A**) Repeated evolution by independent mutations. A unique mutation within each island results in independent evolution of ecotypes within each island. Ecotypes residing on the same island will be closely related at both neutral and adaptive loci. (**B**) Colonization of polymorphic individuals. Admixture between ecotypes within an island results in polymorphic individuals that colonize other islands. Divergent selection on these polymorphisms results in repeated within-island evolution of both ecotypes. (**C**) Adaptive introgression from rare immigrants. Colonization of a single or few individuals of the alternative ecotype that hybridize with the resident ecotype results in adaptive introgression of the alternative alleles. At neutral loci, introgressed haplotypes will be lost by drift due to their low frequency. At adaptive loci, selection may maintain introgressed haplotypes and drive repeated evolution of both ecotypes. (**D**) Colonization between islands, followed by admixture within islands. If reproductive isolation between ecotypes is incomplete, gene flow within islands may result in a closer relationship between species from the same island at neutral loci. At adaptive loci, introgression is reduced due to divergent selection and maintains the close relationship of ecotypic similar species from different islands. (**E**) Colonization between islands. If both ecotypes are reproductively isolated, populations from the same ecotypic species will be closely related at both neutral and adaptive loci.

Independent evolution within islands presumes that the alleles subject to selection evolved independently through unique mutations within each island (Fig. 1A) (*15*, *16*). Yet, an increasing number of studies demonstrate that recurrent ecological differentiation within a radiation often involves repeated selection on the same alleles (*17*–*21*). This suggests that a shared evolutionary history and colonization between islands may drive these repeated divergences (*14*). Three different scenarios, varying in the amount of colonization between islands, can explain a shared history of alleles. First, adaptive alleles could be introduced from a nearby island by colonizing individuals that carry polymorphisms at adaptive loci (“transporter hypothesis”; Fig. 1B) (*22*). Subsequent selection on the alleles at these loci may then result in rapid within-island evolution of similar ecotypes. Second, alleles involved in ecotypic differentiation could be introduced more directly into the gene pool of a resident ecotype when a few individuals of the alternative ecotype colonize the island and hybridize with the prevalent resident ecotype (“adaptive introgression”; Fig. 1C) (*23*). Third, if a larger number of colonizers found a new ecotypic population and hybridize with the resident species, admixture with the resident ecotype may erase the initial genetic differences between these lineages while maintaining differentiation at loci involved in ecotypic differentiation (Fig. 1D). Under this latter scenario, the repeated occurrence of ecologically similar species on the different islands primarily involves colonization of ecotypes between islands (Fig. 1E) and no within-island diversification is actually involved (*11*). Although these scenarios differ substantially in the contribution of evolutionary (within-island diversification) versus ecological (between-island colonization) processes in the establishment of phenotypic similar sets of species on the different islands, patterns of genetic differentiation can exhibit remarkable similarities (Fig. 1) (*24*, *25*). This has questioned the degree of support for parallel evolution in insular systems for even some of the most iconic examples of adaptive radiation (*11*, *13*). Identifying the genetic variants underlying repeated island radiations and reconstructing their evolutionary history is therefore essential. However, the current scarcity of genomic data from insular radiations limits our understanding of this issue (*26*).

**Fig. 2.**
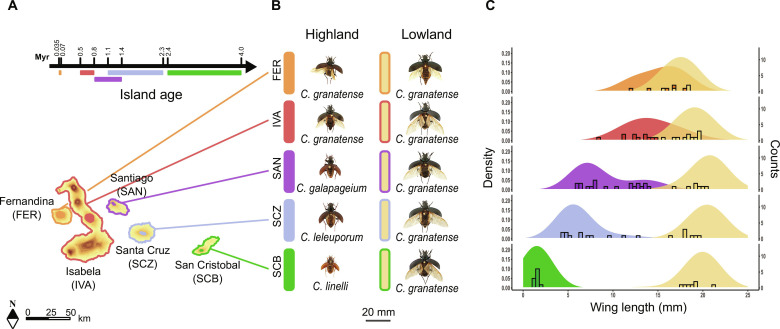
Geographic distribution and wing lengths of *Calosoma* beetles at the Galápagos islands. (**A**) Ages (*36*) of the sampled Galápagos islands. Colored regions show the approximate distribution of the highland species *C. linelli* (green), *C. leleuporum* (light blue), *C. galapageium* (purple), the *C. granatense* highland populations of Volcan Alcedo on Isabella (IVA) (red) and Fernandina (FER) (orange), and the lowland populations of *C. granatense* (yellow). Myr, million years. (**B**) Pictured specimens of all sampled *Calosoma* species and populations, ordered from the youngest (upper) to oldest (lower) islands. SCB, San Cristobal; SCZ, Santa Cruz; SAN, Santiago. (**C**) Density distribution of the wing lengths based on (*27*). Bars show wing lengths of individuals used for genomic analysis in this study.

Here, we use genome-wide genetic variation to reconstruct the relative contribution of ecological (i.e., colonization between islands) and evolutionary (i.e., diversification within islands) processes to the parallel evolution of caterpillar hunter beetles (*Calosoma* sp.) to elevation gradients along the Galápagos archipelago (Fig. 2) (*27*, *28*). The radiation consists of repeated and gradual highland adaptation along the progressive age of the Galápagos islands, and we use the different island ages, associated with different stages of parallel divergence, as a powerful tool to infer the role of historical processes and the source of adaptive alleles involved in ecotypic divergence (*29*). At low elevations, *Calosoma granatense*, a single long-winged species capable of dispersal by flight (*30*), is found on all major islands. However, high elevations of the old and intermediate-aged islands San Cristobal, Santa Cruz, and Santiago are each occupied by a distinct highland species, taxonomically described as *C. linelli*, *C. leleuporum*, and *C. galapageium*, respectively (Fig. 2, A and B). These highland species share morphological traits such as a marked reduction in wing size, which likely evolved through selection for reduced dispersal in the more stable highland habitats (*31*, *32*), but the degree of morphological divergence from the lowland species decreases toward more recent islands (Fig. 2C). On Santiago, putative hybrid individuals with intermediate phenotypes have been found, indicating that reproductive isolation between at least *C. galapageium* and *C. granatense* is still incomplete (*28*). Highlands of the youngest islands Isabela and Fernandina are occupied by populations of the lowland species *C. granatense* that evolved a wing size reduction in line with the divergence of the highland species on the older islands (Fig. 2C).

By characterizing the loci involved in highland adaptation, we leverage the progressive divergence to test if high- and lowland species evolved independently on each island or if this is driven by an ancient, initial, high-lowland divergence followed by the exchange of genetic variation between islands. By showing that highland species evolved through selection of alleles whose origin coincided with the most ancient high-lowland divergence within this radiation and subsequently spread progressively toward more recent islands, we demonstrate how an initial singular ecotypic divergence and a mixed contribution of colonization between islands and evolution within islands contribute to the emergence of parallel species assemblages in insular systems.

## RESULTS

### Repeated ecotype divergence strongly correlates with island age

**Fig. 3.**
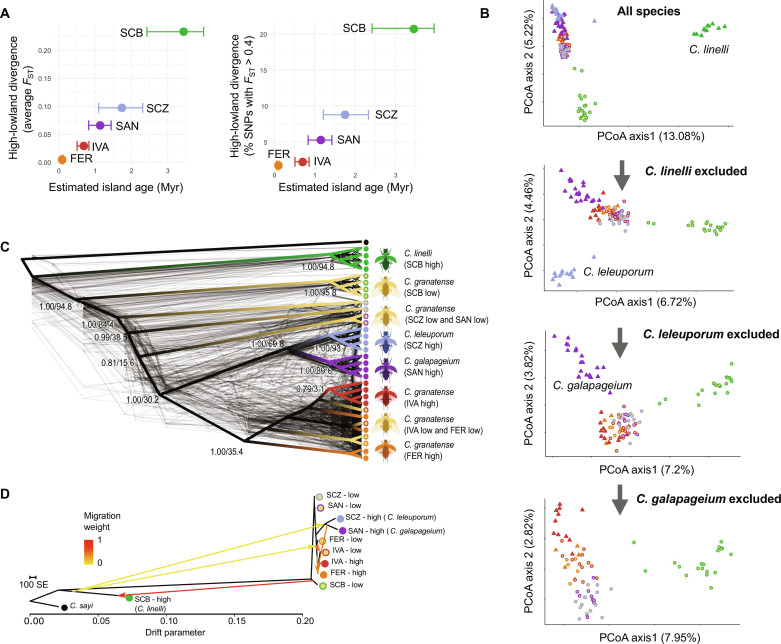
Genetic differentiation and whole-genome phylogenetic relationships between the *Calosoma* species and populations from the Galápagos. (**A**) Relationship between island age and within-island high-lowland divergence (left panel: average *F*_ST_; right panel: percentage of SNPs with *F*_ST_ > 0.4) and the estimated age of the island. Estimated ages of each island are based on (*36*). (**B**) PCoA of the species and populations based on SNP data obtained from RADtag sequencing. The most divergent highland species was sequentially excluded toward bottom panels. (**C**) Whole-genome phylogenetic relationship between the species and populations as inferred from 105 ML trees obtained from nonoverlapping genomic windows of 1 Mb (light gray trees), with the multispecies phylogeny [ASTRAL (*76*)] superimposed. Branch labels show branch support values before slash and gCFs (*77*), which express the percentage of ML trees containing this branch after the slash. (**D**) Population tree and population admixture (arrows) of the different *Calosoma* populations and species, based on SNP data obtained from RADtag sequencing, using TreeMix with six migration edges. Color codes of the different species and populations in all panels are as in Fig. 2. Genomic regions involved in ecotypic high-lowland divergence were excluded for the analyses presented in (B) and (C).

We assessed patterns of genetic differentiation between high- and lowland species from the major islands by mapping 1135 restriction site–associated DNA sequence tags (RADtags), obtained from 5 to 22 individuals per population, to a newly assembled genome of the lowland species (data S1 to S3). Average genetic differentiation (*F*_ST_) between high- and lowland populations was consistent with the patterns of morphological divergence and increased almost linearly toward older islands (*r*_S_ = 1, *P* = 0.017; Fig. 3A and data S4). This increase in genomic high-lowland differentiation was primarily identified as an increased frequency of single-nucleotide polymorphisms (SNPs) with high *F*_ST_ values. For example, the proportion of highly differentiated SNPs (*F*_ST_ > 0.4) in the within-island high-lowland comparison increased consistently from 2% on the most recent island Fernandina to 20% on the oldest island San Cristobal (Fig. 3A). Both a Bayesian clustering approach and a principal coordinates analysis (PCoA) on neutral SNP genotypes confirmed this gradual differentiation of highland species along the island progression, with the highland species from the oldest island San Cristobal (*C. linelli*) being the most divergent species, followed by the highland species from Santa Cruz (*C. leleuporum*) and subsequently Santiago (*C. galapageium*) (Fig. 3B and Supplementary Text).

### Phylogenetic incongruencies suggest admixed species histories

We inferred the phylogenetic relationship among the different species and populations using whole-genome resequencing data from at least four individuals per species and reconstructed maximum likelihood (ML) trees from consecutive windows of 1 Mb. We excluded windows with extensive regions showing elevated divergence between the ecotypes to ensure that phylogenetic relationships were based on neutral genetic variation and not influenced by potential sharing of adaptive alleles among species belonging to the same ecotype. Both individual ML trees and the species tree, which integrates these individual trees, supported some basal relationships that were largely congruent with the patterns of genetic differentiation (Fig. 3, B and C, and data S4). Our phylogenetic analysis supported the strong divergence between the highland species of the oldest island San Cristobal (*C. linelli*) and all other species, marking this as the initial divergence within this radiation. Subsequently, the lowland population from this oldest island (*C. granatense*) diverged from the remaining species and *C. granatense* populations. This is followed by divergence of the *C. granatense* lowland populations from the subsequent oldest islands Santa Cruz and Santiago (Fig. 3C), although their exact position within this clade appeared less clear. Moreover, individuals from these *C. granatense* populations depicted secondary shallow relationship with the *C. granatense* populations from the most recent islands Isabella and Fernandina, indicating that substantial gene flow among *C. granatense* populations from the different islands, except San Cristobal, took place after their initial divergence. The species phylogeny further strongly supported a shared common ancestry of the highland species of Santa Cruz (*C. leleuporum*) and Santiago (*C. galapageium*). PCoA confirmed the shared genetic variation between the highland species from these two islands as demonstrated by their similar position along PC1 but, in addition, indicated that differences in SNP allele frequencies between the lowland species *C. granatense* and *C. galapageium* tend to be smaller than those between *C. granatense* and *C. leleuporum* (PC2, Fig. 3B). These patterns were corroborated by pairwise *F*_ST_ estimates (data S4). This suggests that the highland species of Santiago originated from the highland species of Santa Cruz but subsequently experienced considerably higher admixture with the lowland species *C. granatense* after colonization, resulting in an apparent pattern of within-island divergence on Santiago based on patterns of genetic differentiation only.

Although most nodes in our multilocus species tree were generally well supported, gene concordance factors (gCFs), indicating the percentage of individual trees containing a particular node, were often low for the different *C. granatense* populations and the clade containing the two highland species *C. leleuporum* and *C. galapageium*. This indicates a substantial sharing of genetic variation between *C. granatense* and highland species of more recent islands and, hence, a relative low genetic integrity for these species.

The tree topology as inferred from TreeMix (*33*) confirmed the strong divergence of the highland species of San Cristobal (*C. linelli*) from the closely related remaining species and populations (Fig. 3D). Congruent with our multispecies phylogeny, the remaining two highland species (*C. leleuporum* and *C. galapageium* of Santa Cruz and Santiago, respectively) grouped in a clade that was situated within the populations of the lowland species *C. granatense*. Both TreeMix and an introgression analysis based on f_4_ statistics (*34*) supported numerous interspecific migration events within as well as between islands (Supplementary Text). Ancestral genetic variation of the distinct highland species of San Cristobal (*C. linelli*) was retained in the *C. granatense* lowland population of this island and in the two other highland species (*C. leleuporum* and *C. galapageium*). Signatures of such ancient admixture could even be traced back to the highland populations of *C. granatense* inhabiting the youngest islands (Fig. 3D, Supplementary Text, and data S5).

### Outlier loci are shared across islands

**Fig. 4.**
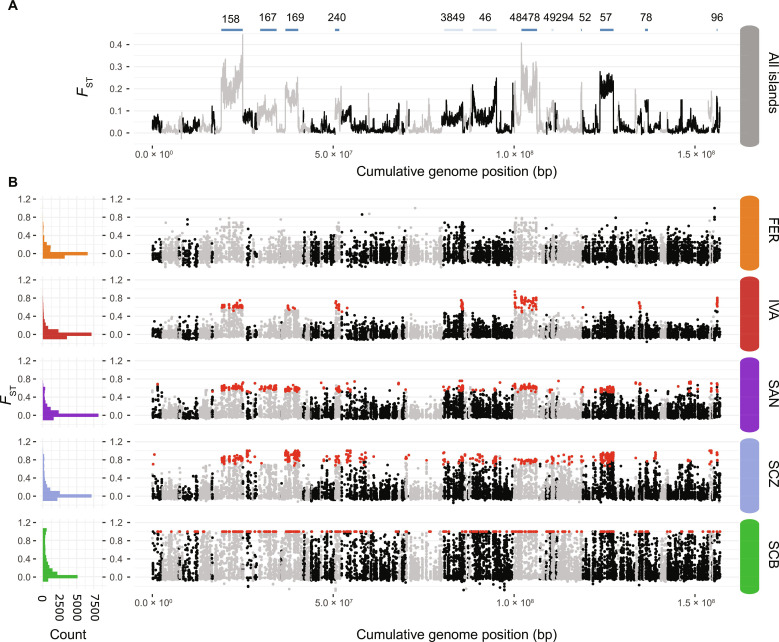
Genetic differentiation between the high- and lowland species or populations. (**A**) Genetic differentiation (*F*_ST_) between all resequenced high- versus lowland individuals (20-kb windows). Upper blue line segments show the location of genomic regions tested for the presence of SVs, with those in dark blue being regions showing support for SVs based on anomalies in the orientation and insert size of read mappings as detected by BreakDancer (*78*). Scaffold IDs are given above each SV. bp, base pair. (**B**) Differentiation (*F*_ST_) between high- and lowland individuals within each island at individual SNPs obtained from RADtag sequencing. Left histograms show the *F*_ST_ frequency distribution across the entire genome, and right panels show their location at the different genomic scaffolds. SNPs indicated in red are outlier SNPs with a significantly higher differentiation than expected by chance within each island comparison [BayeScan (*35*); *Q* value <0.1]. Color codes are as in Fig. 2.

Within islands, we identified the genomic regions associated with high-lowland divergence by screening the RADtag sequences for SNPs that were significantly more differentiated compared to background levels in the within-island comparisons [BayeScan (*35*), *Q* value <0.1]. We identified between 196 (Isabella) and 458 (Santiago) outlier SNPs in these separate within-island comparisons, except for the most recent island Fernandina for which a lower number of individuals could be sampled (Fig. 4B). These outlier SNPs were not randomly distributed across the genome but generally clustered into large genomic blocks extending up to several megabases (Fig. 4B). Genomic regions characterized by an elevated divergence in the high-lowland comparison within each island were highly consistent across the different islands, indicating that largely the same genomic regions are involved in each high-lowland divergence on the different islands.

### Outlier loci include extensive chromosomal inversions

**Fig. 5.**
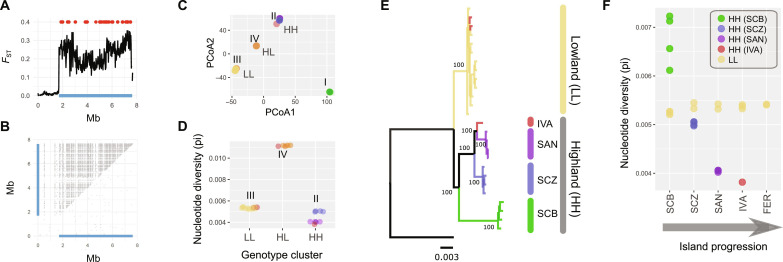
SVs underlie distinct alleles associated with high-lowland divergence. Results of a single scaffold (scaffold158) are shown. Results for the other scaffolds with SVs associated with high-lowland divergence are shown in fig. S4. (**A**) *F*_ST_ distribution (20-kb windows) based on a comparison between all resequenced high- versus lowland individuals. Blue bottom line shows the location of the chromosomal rearrangements detected by BreakDancer (*78*). Red dots show the location of RADtags identified as outlier loci in at least two within-island highland-lowland comparison. (**B**) Location of SNPs in perfect linkage disequilibrium (*r*^2^ = 1). Gray dots above the diagonal show *r*^2^ = 1 values for all 32 resequenced individuals. Gray dots below the diagonal show *r*^2^ = 1 values for homozygous lowland individuals only. (**C**) PCoA based on SNPs located at the inversion [blue line in (A)]. HH (cluster II), LL (cluster III), and HL (cluster IV) refer to the cluster of individuals genotyped at the inversion as homozygous for the highland allele (HH) and lowland allele (LL) and heterozygous (HL), respectively. Highland alleles of San Cristobal are denoted as cluster I. (**D**) Differences in nucleotide diversity at the inversion between individuals genotyped as HH, HL, and LL in (C). (**E**) ML tree of the nucleotide sequence at the inversion. Individuals genotyped as heterozygotes (HL) were excluded from the analysis. Node values represent bootstrap values based on 1000 replicates. The tree was rooted with the mainland species *C. sayi*. (**F**) Relationship between individual nucleotide diversity at the inversion and the progression of the islands. Only individuals genotyped as homozygous for the lowland allele (yellow) and highland allele (remaining colors) are included. Color codes are as in Fig. 2.

Because most outlier SNPs were concentrated into large genomic blocks that were shared across islands, we tested if the alleles under divergent selection potentially included structural variations (SVs). We investigated the presence of such SVs for the 12 longest contiguous genomic regions with elevated divergence (*F*_ST_ > 0.1) in an overall high-lowland comparison (Fig. 4A). An SV analysis based on anomalies in the orientation and insert size of read pairs identified chromosomal inversions that perfectly overlapped with the five largest (3.7 to 5.9 Mb) and one smaller (213 kb) regions of elevated divergence (Fig. 5A, fig. S4, and data S6). Two additional regions of elevated divergence included the scaffold start and read pairs situated at the potential SV breakpoint mapped to another scaffold and thus likely represent partially assembled inversions. One last region was flanked by deletions and potentially comprises a more complex SV. Three remaining regions did not show evidence for SV based on anomalous read mappings at the flanking regions (data S6).

SNPs located on each SV generally showed an identical segregation pattern with no obvious decay in linkage disequilibrium across the entire length of the SV (Fig. 5B and fig. S4), providing additional support that extensive nonrecombining and highly divergent haplotypes underlie these regions of elevated divergence. A local PCoA based on the SNP genotypes within each SV consistently clustered individuals into four distinct groups (Fig. 5C and fig. S4): (I) a cluster of individuals from the highly divergent species *C. linelli*; (II) a cluster of individuals from the highland species *C. leleuporum*, *C. galapageium*, and the highland populations of *C. granatense*; (III) a cluster that mainly comprised individuals from the lowland species *C. granatense*; and (IV) a cluster containing individuals of both high- and lowland species and populations that was situated in-between clusters II and III. This clustering is consistent with the presence of a distinct high- and lowland allele, with clusters II and III comprising individuals homozygous for the high- and lowland allele, respectively, and cluster IV corresponding to individuals being heterozygous for both alleles. Heterozygosity for a distinct high-and lowland allele in the individuals of this latter cluster (IV) was supported by a markedly higher nucleotide diversity at the SV compared with those homozygous for one of the two alleles (clusters II and III, Fig. 5D). Moreover, these elevated levels of nucleotide diversity in heterozygotes were maintained across the entire length of the SV, which provides additional support for the lack of recombination among those divergent haplotypes (fig. S5).

### Progressive spread of inversions that originated from an initial high-lowland divergence

To infer the evolutionary history of the chromosomal inversions, we constructed ML phylogenies of the haplotypes present at each SV. Haplotypes associated with all highland species and populations, including the highly distinct haplotypes of the most divergent highland species *C. linelli* from San Cristobal, consistently clustered with high support into a monophyletic clade (Fig. 5E and fig. S4). Thus, SV haplotypes selected in all highland species and populations appear to have a single evolutionary origin and subsequently spread across all islands. Spread of highland alleles generally followed the island progression as shown by a consecutive split of highland haplotypes according to island age for six SVs (Fig. 5E and fig. S4). This progressive spread was further corroborated by a significant and consistent decrease in nucleotide diversity of highland alleles toward younger islands for all but one SV (Fig. 5F, fig. S4, and data S6). The highly consistent phylogenetic and nucleotide diversity patterns across the different SVs could, at least partially, be caused by a tight physical linkage of the scaffolds on which the SVs are located. However, none of the SVs showed an identical segregation pattern across the 32 investigated individuals, which demonstrates that they represent independently evolved loci with a shared evolutionary history (fig. S6).

**Fig. 6.**
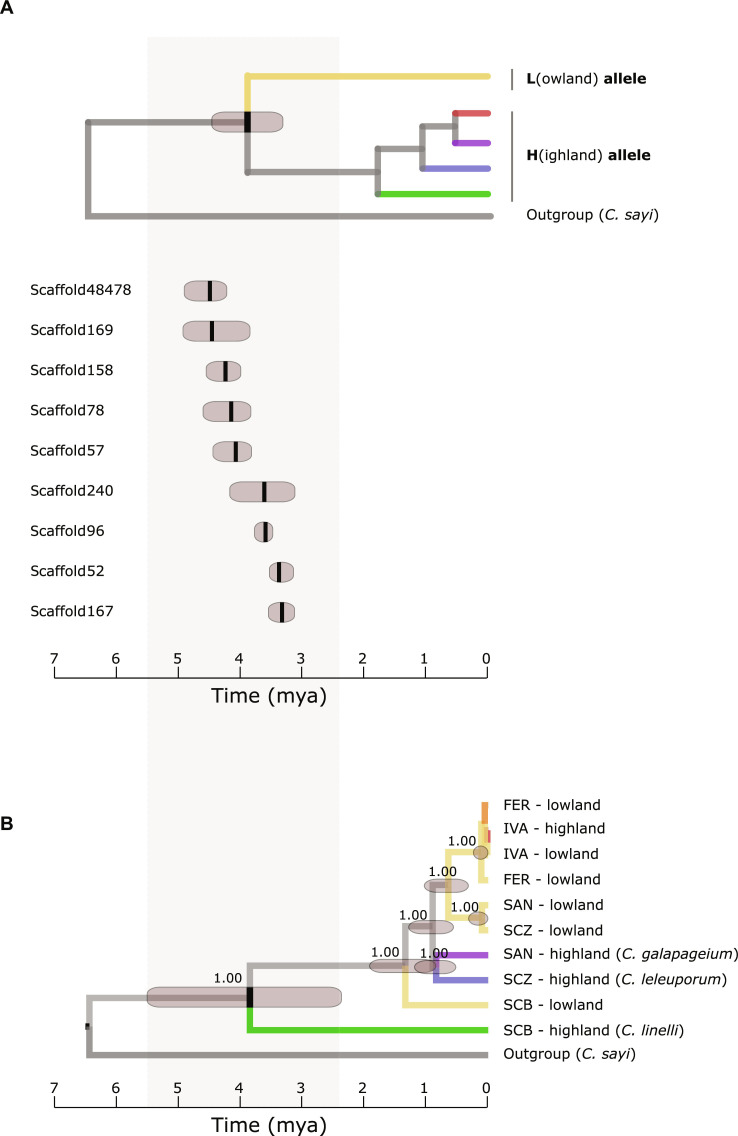
Estimated divergence time between high- and lowland alleles for nine different SVs compared to the estimated divergence time between the species. (**A**) Estimated divergence time (black vertical bars: mean; dark gray bars: 95% highest posterior density) between high- and lowland alleles for nine different SVs associated with high-lowland divergence. (**B**) Estimated divergence times of the different species and populations based on random selection of 50 genomic windows of 20 kb that are located outside the SV. Node values represent posterior probabilities of the clades. Divergence times in both panels are expressed in mya using the divergence from the most closely related extant mainland species *C. sayi* as calibration point.

Last, we investigated if the divergence between the high- and lowland associated alleles across all SVs evolved during a singular high-lowland divergence event by comparing their timing of the split between high- and lowland alleles (Fig. 6A). Estimated divergence times ranged between 3.3 and 4.5 million years ago (mya), roughly corresponding to the estimated emergence time of the oldest extant island San Cristobal, and 95% posterior density intervals of the splitting times between high- and lowland selected alleles overlapped for six of the nine SVs. These estimated divergence times were further centered around the estimated divergence time of the most ancient high-lowland divergence in our species phylogeny at 3.84 mya, being the split that gave rise to the San Cristobal highland species *C. linelli* and the remaining species, including the lowland species *C. granatense* (Fig. 6B). This suggests that the evolution of highland alleles coincides with the most ancient high-lowland divergence in this radiation.

## DISCUSSION

Archipelagos where islands emerged following a known chronosequence have proven key to reconstruct the processes of species diversification (*2*, *3*, *9*). In particular, when radiations within these island groups result in the repeated evolution of similar forms, these systems provide a unique opportunity to explore the mechanisms that shape deterministic evolutionary trajectories. Using this Galápagos beetle radiation where highland species and populations have gradually and repeatedly evolved similar traits along the island progression, we investigated if an ancestral divergence and subsequent exchange of genetic variation and individuals may drive these repeated divergences. Patterns of genome-wide divergence corroborated a repeated evolution of distinct highland species and populations within the different islands by showing a gradual decrease in differentiation from the lowland species toward younger islands. However, adaptation toward highland habitats involved selection within the same genomic regions across all highland species. These genomic regions were characterized by extensive chromosomal inversions, often extending multiple megabases, that resulted in distinct and nonrecombining haplotypes associated with high-lowland divergence. The well-supported monophyletic clustering of highland associated haplotypes provides strong evidence that a single evolutionary origin underlies the highland alleles at each locus. Moreover, high- and lowland selected alleles across loci diverged within a similar time frame, corresponding with the emergence time of the oldest island San Cristobal and the estimated divergence time of its highland species *C. linelli*. Thus, the repeated evolution of highland species and more recent highland populations appears driven by selection on alleles that evolved during a singular high-lowland divergence event that coincided with the most ancient high-lowland species divergence within the archipelago.

The phylogenetic pattern of haplotypes that were associated with highland habitats depicted consecutive splits consistent with the chronosequence of the islands. This consistent phylogenetic signal was likely preserved by the association of highland haplotypes with reduced dispersal capacity, which increased their geographic isolation and prevents their subsequent exchange between the islands (*32*). Combined with a steady decrease in nucleotide variation toward younger islands, consistent with serial and stepwise founder events of highland alleles, this pattern supports a progressive spread of highland alleles along this island progression. Propagation of highland alleles toward more recent islands might either have occurred through (i) colonization of highland species between islands (Fig. 1D), (ii) colonization of a few highland individuals that introduced highland alleles in resident lowland populations (“adaptive introgression”; Fig. 1C) (*23*), or (iii) colonization of lowland individuals that are polymorphic at loci involved in high-lowland divergence (Fig. 1B). Our data suggest that several of these mechanisms took place within this radiation. Colonization of more recent islands by highland species was supported by the sister relationship of the highland species of Santa Cruz (*C. leleuporum*) and Santiago (*C. galapageium*) in our phylogenetic analysis, which indicates that the highland species of Santiago evolved from highland individuals of Santa Cruz that colonized Santiago, rather than through a high-lowland divergence within Santiago. This appears plausible given the connection between both islands about 1 mya (*36*). Although our genome-wide phylogeny revealed that 70% of the genomic windows (gCFs) support the sister relationship between these highland species, considerable gene flow with the lowland species *C. granatense* took place after the colonization of Santiago and partially erased this ancestral phylogenetic signature of the sister relationship between those two highland species. In contrast, spread of highland alleles by colonization of polymorphic individuals of the lowland species *C. granatense* took place between, at least, the more recent islands Isabela and, particularly, Fernandina. For these islands, we found that winged lowland individuals of *C. granatense* are highly polymorphic at loci under divergent selection (fig. S6), which may easily result in the transport of highland alleles between islands. Colonization of “pure” highland individuals between these islands can moreover be excluded as highland habitats of these more recent islands are not populated by distinct highland species but by individuals of the lowland species with reduced wings and a higher frequency of highland alleles.

While decreasing levels of ecotypic divergence along a chronosequence is often considered to represent different stages of the speciation process (*29*, *37*), our results rather point toward a reversal of the high-lowland divergence along this island progression. More precisely, while high- and lowland species are morphologically and genetically well differentiated on the oldest island San Cristobal, their divergence decreases toward the younger islands Santa Cruz and Santiago. Provided the sister relationship of the highland species on these latter two islands, this lower genomic divergence from the lowland species on Santiago implies increased high-lowland admixture on this younger island (Fig. 1D). Increased admixture between high- and lowland species on more recent islands may ensue from the stepwise colonization of highland species on islands with a resident lowland population. This leads to decreasing founder population sizes of highland species on more recent islands, which, in turn, increases asymmetric introgression from the large population of the resident lowland species *C. granatense* into the smaller colonizing population of highland individuals (*38*, *39*). If the number of immigrant highland individuals becomes very small, then these individuals may even be more likely to exchange genes with the resident lowland population rather than with other highland immigrants, resulting in adaptive introgression (*23*) of highland alleles in the resident lowland population (Fig. 1C). Divergent habitat-mediated selection may maintain these introgressed alleles within highland populations, which may then ultimately result in the repeated evolution of the highland ecotype. Because introgression of adaptive alleles leads to polymorphisms at adaptive loci, highland alleles may ultimately be introduced by the immigration of such polymorphic individuals (Fig. 1B), a process that likely took place at the youngest islands Isabela and Fernandina of the archipelago. Therefore, patterns of divergence along this island progression provide unique support and an extant illustration of the presumed stages of the emerging “two-time frame” model of repeated ecotype evolution, which proposes that contemporary ecotypic evolution is driven by selection on alleles that potentially originate from an old, singular, and even allopatric ecotypic divergence (*18*, *19*).

The parallel occurrence of ecological and morphological similar species pairs on islands is presumed to result either from the colonization of species between islands or through repeated adaptation to different environments within islands (*2*). Results from our study demonstrate that complex introgression patterns between and within islands challenge this dichotomous view and suggest that the difference between these two mechanisms is likely more nuanced than generally assumed. Habitat patches on islands are highly dynamic with respect to their spatial and temporal configuration, often driven by climatic and geological dynamics, resulting in multiple episodes of fission and fusion between diverging populations (*9*, *40*). This may both erase the original phylogenetic signals of species divergence and result in the exchange of alleles involved in ecotypic differentiation between islands. Chromosomal rearrangements could strongly facilitate the repeated evolution after adaptive introgression and resist the effect of homogenizing gene flow by maintaining favorable allelic combinations (*41*–*45*). Given the frequently reported evidence of interspecific gene exchange in island radiations such as, for example, Darwin’s finches (*46*, *47*), *Anolis* lizards (*48*), giant tortoises (*40*, *49*), *Hogna* wolf spiders (*50*), Hawaiian silverswords, lobelioids (*51*), and *Metrosideros* trees (*20*), these complex introgression patterns can be expected to be ubiquitous. The number of founding individuals or haplotypes of the alternative ecotype that colonize an island and the amount of interspecific gene flow between the two ecotypes after colonization are likely key factors that determine the relative contribution of inter-island colonization and within-island diversification in the origin of parallel species assemblages on islands. Better comprehending the interplay between these mechanisms could help to better predict how rates of colonization and speciation determine biodiversity dynamics on islands (*14*, *52*).

## MATERIALS AND METHODS

### Sampling

We sampled all high- and lowland species or populations from all islands for which distinct highland ecotypes or species have been reported at the Galápagos archipelago, i.e., San Cristobal, Santa Cruz, Santiago, Isabela, and Fernandina (data S1 and Fig. 2). The island Isabela is the only island within the Galápagos that comprises multiple large volcanoes, and we restricted our sampling to the most centrally located Volcan Alcedo. Individuals were sampled during different sampling campaigns between 1996 and 2014 (data S1) and stored live in liquid nitrogen or pure ethanol shortly after sampling. While high- and lowland species are easily identified in the field on the oldest islands San Cristobal, Santa Cruz, and, to a lesser extent, Santiago (*27*), divergence of highland ecotypes varies more gradually on the younger islands Isabela and Fernandina. To ensure that individuals of the highland ecotype were sampled at these islands, only those individuals sampled at the outermost volcano summit (1110 and 1290 m at Isabela and Fernandina, respectively) were considered as highland ecotypes. We included between 11 and 22 individuals per population for genetic analysis, except for Fernandina where only 5 individuals of the high- and lowland population could be sampled. Wing sizes of the sampled individuals clearly matched the earlier reported wing sizes of these species and populations (Fig. 2, B and C) and confirmed the gradual reduction in wing size of the highland species and populations toward younger islands (*30*). For the most recent island Fernandina, wing sizes of highland individuals overlapped with those from lowland individuals. We further sequenced the genome of a single specimen of the related mainland species *C. sayi* (*53*, *54*), sampled by M. Husemann in Texas, United States, and was used as an outgroup species. *C. sayi* is one of the most closely related species with those found at the Galápagos and taxonomically classified within the same subgenus *Castrida* (*55*).

### Genome assembly

We assembled the genome of *C. granatense* using both paired-end libraries with short insert sizes of 170, 500, and 800 bp and mate-paired libraries with insert sizes of 2, 5, 10, and 20 kb. Short–insert size libraries were all constructed from a single individual sampled at Santa Cruz at a 350-m altitude, while long-insert mate paired libraries were constructed from DNA extracts from nine different individuals that all originated from this same locality (data S1). Total DNA was extracted from these individuals with the NucleoSpin Tissue Kit, Macherey-Nagel GmBH, and library construction and sequencing were performed at the Bejing Genomic Institute, Hong Kong. Sequencing errors were corrected based on the *k*-mer frequency spectrum with SOAPec (*56*), specifying a *k*-mer value of 17. Corrected reads were then used as input for genome assembly with Platanus (*57*) using default settings. Contigs were constructed based on the short-insert libraries only with the “platanus assemble” tool and subsequently combined into scaffolds with the “platanus scaffold” tool using both short- and long-insert libraries. Gaps between the scaffolds were lastly filled with the “platanus gap_close” tool using both short- and long-insert libraries. The final assembly consisted of 6045 scaffolds summing to a size of 167,880,245 bp (data S3). We estimated the genome size by obtaining the *k*-mer frequency spectrum from whole-genome sequencing data from multiple individuals (see the "Restriction site–associated sequencing and whole-genome resequencing" section) with Jellyfish v2.3.0 (*58*) and analyzed the frequency distribution with GenomeScope (*59*). These analyses yielded an estimated genome size of 173.3 Mb (± 6.0SD) for *k*-mer = 21 and highly similar estimates for other tested *k*-mer sizes (*k*-mer = 17: 172.6 Mb ± 8.2SD; *k*-mer = 31: 172.6 Mb ± 8.2SD). On the basis of these estimates, the assembled genome represents 97% of the estimated genome size. Completeness of the assembly was further assessed based on a set of 1658 benchmarked single-copy orthologs (BUSCOs) from Insecta (*60*). Screening the draft genome for these BUSCOs revealed that 85.3% were present in our assembly, of which 0.3% duplicated and 8.4% fragmented (data S3). We screened the genome for repetitive elements with RepeatMasker v1.295 (*61*) specifying “Coleoptera” as species and constructed a library of de novo repetitive elements with RepeatScout v1.0.5 (*62*). Both methods resulted in a total repeat content of 11.04% (data S3).

### Restriction site–associated sequencing and whole-genome resequencing

We performed restriction site–associated sequencing (RADseq) on between 11 and 22 individuals of each population, except for the island of Fernandina where only 5 individuals of the high- and lowland population were available (data S1). DNA was extracted using the NucleoSpin Tissue Kit, Macherey-Nagel GmBH following the manufacturer’s instructions. DNA extracts were normalized to a concentration of 7.14 ng/μl, and RADtag libraries were constructed following the protocol described in (*63*) using the SbfI-HF restriction enzyme (NEB) and sequenced on either an Illumina MiSeq (2x250bp) or HiSeq1500 (2x100bp) platform. Raw data were demultiplexed to individual samples using the *process_radtags* module in Stacks v1.20 (*64*). polymerase chain reaction duplicates were removed with the *clone_filter* tool based on identical reverse read ends. Paired reads were mapped to the draft reference genome with the Burrows-Wheeler Alignment tool (bwa mem) (*65*) using default settings, and SNPs were called using GATK’s UnifiedGenotyper tool. Only biallelic SNPs (--max-alleles 2) with a minimal SNP quality (--minQ) of 60, an individual genotype (--minGQ) quality of 30 in at least 80% of the individuals (--max-missing), and a minimum allele frequency (--maf) of >0.05 were retained with VCFtools (*66*). We further excluded all positions located in repetitive regions detected by RepeatMasker v1.295 (*61*) (see the “Genome assembly”section). After filtering, we retained 15,256 SNPs.

We further sequenced the genomes of 33 individuals comprising four individuals of each highland species and population, four individuals of the *C. granatense* lowland population of San Cristobal, two individuals of the remaining *C. granatense* lowland populations, and an individual of the related species *C. sayi* from Texas, United States (data S1 and S2). Genomic libraries were constructed with the TruSeq Nano DNA LT kit (Illumina) following the manufacturer’s instructions and sequenced on an Illumina HiSeq1500 platform (2x100bp). Resulting sequencing reads were mapped to the draft reference genome with the Burrows-Wheeler Alignment tool (bwa mem) (*65*) with default settings. Local indel realignment was performed using GATK’s RealignerTargetCreator and IndelRealigner (*67*). Variants were first called for each individual sample using GATK’s HaplotypeCaller and subsequently called across all samples with the GenotypeGVCF tool. Variants were lastly hard filtered with the VariantFiltration tool specifying the following five criteria: quality score normalized allele depth (QD) < 2.0, FisherStrand (FS) > 60.0, MappingQuality (MQ) < 40, Mapping Quality Rank Sum (MQRandSum) < −12.5, and ReadPosRankSum < −8.0 and removed SNPs located in repetitive regions. A total of 15,569,155 SNPs were retained after filtering, of which 98% were shared by at least 80% of the resequenced individuals.

### Patterns of genomic divergence

We estimated genome-wide genetic differentiation between the high- and lowland populations within each island based on the *F*_ST_ values of SNPs obtained from our RADseq data (1135 RADtags). To further explore the neutral genetic population structure, we subsequently removed RADtags located in genomic regions of elevated between-ecotype divergence (*F*_ST_ > 0.1) (see the “SV analysis” section) and RADtags containing an outlier SNP in at least two within-island ecotype comparisons (see below), which resulted in a subset of 900 RADtags. Pairwise mean *F*_ST_ values were calculated for each population/species pair using VCFtools v0.1.16 (*66*). Next, we ran a PCoA using the R package adegenet 2.1.3 (*68*). To minimize linkage disequilibrium between SNPs, we randomly selected a single SNP within each neutral RADtag (*n* = 900) using an in-house Python script. We sequentially discarded the most diverged highland species in subsequent PCoAs to further reveal the hierarchical population structure and to explore the relationships among the remaining clusters in more detail. Last, we used the identical dataset to perform an individual-based Bayesian admixture analysis implemented in STRUCTURE 2.3.4. (*69*) to assess the number of distinct genetic clusters (*K*) and level of admixture among genetic lineages. Models were fitted with 10 independent replicate runs for each *K* = 2 to 8 using 100,000 Markov chain Monte Carlo repetitions with a burn-in period of 30,000, correlated allele frequencies, and no prior information on the population of origin. All other default setting were retained.

To assess if SNPs within our 1135 RADtags were a putative target of natural selection, we evaluated if among-population genetic differentiation was significantly higher than expected under neutrality using BayeScan v2.1 (*35*). This outlier analysis calculates the posterior probability of each SNP to be the target of selection by contrasting a model that includes the effect of selection to one excluding such effect. Simulations were performed using a total of 20 pilot runs of 5000 iterations each to tune model parameters. Subsequently, we ran the Markov chain Monte Carlo for another 100,000 iterations, discarded the first 50,000 as a burn-in while setting the prior odds for the neutral model to 10, and used the internal *q* value function of the software package to assess significance at a false discovery rate threshold of 0.1 (*q* < 0.1).

Genetic differentiation between all resequenced high- and lowland individuals was assessed using a sliding window approach to minimize noise from SNP-based divergence estimates. Weir and Cockerham’s *F*_ST_ statistics (*70*) were estimated for nonoverlapping 20-kb windows using VCFtools v0.1.16 (*66*).

### Phylogenetic analysis

We inferred the phylogenetic relationship between the resequenced individuals across the entire genome based on nonoverlapping genomic windows of 1 Mb each. Windows located at the end of scaffolds or located on scaffolds with a length smaller than 1 Mb were only included if their length measured at least 0.5 Mb. To ensure that the phylogenetic relationships reflected neutral patterns of species divergence and were not driven by sharing of adaptive alleles among ecotypes, 50 windows comprising genomic regions with elevated divergence (*F*_ST_ > 0.1) in an overall high-lowland divergence were excluded (see the “SV analysis” section), resulting in a final set of 105 windows used for phylogenetic analysis. Fasta files containing the individual sequences for each window were then extracted with the vcf2fasta.pl tool (https://github.com/santiagosnchez/vcf2fasta), using the genome assembly, genome-wide VCF, and gff file specifying the locations of the 1-Mb windows as input files. We estimated ML trees for each window-specific fasta with IQ-TREE (*71*), specifying 1000 ultrafast bootstrap samplings (*72*). Before tree estimation, best substitution models for each window were selected using ModelFinder (*73*) as implemented in IQ-TREE. ML trees were visualized with the densiTree function implemented in the *phangorn* v2.5.5 (*74*) package in R v4.0.3. To ease visualization, trees were first made ultrametric by transforming the branches proportionally in FigTree v1.4.2 (http://tree.bio.ed.ac.uk/software/figtree). We then reconstructed a multispecies phylogeny that accounts for the potential discordance in the window-specific phylogenies using ASTRAL (*75*, *76*). Besides the calculation of branch support values as implemented in ASTRAL (*76*), we also calculated gCFs (*77*), which express the percentage of gene (window) trees containing this branch. gCFs were obtained from IQ-TREE by specifying the multispecies consensus tree from ASTRAL as reference tree.

We applied a graph-based model implemented in TreeMix v1.13 (*33*) to explore evolutionary relationships and admixture events among resequenced high- and lowland populations and species. A maximum-likelihood tree was initially inferred, after which the inclusion of 1 to 10 gene flow events (-*M*) between different populations and species was allowed to improve model fit (Supplementary text). Such migration events represent either population admixture or shared ancestral polymorphism retained after population isolation. For each setting, the model was ran for 10 iterations while sites were pooled into blocks of 500 SNPs (-*k* 500) to account for linkage disequilibrium. The tree was rooted using *C. sayi*, and only SNPs without missing population allele frequencies were included (*n* = 12,321,150) to minimize bias in variance-covariance matrix estimation. To delineate the optimal number of migration events, we compared the mean log likelihood of a model with that of a model containing one additional migration event using a *t* test. Initiating at *M* = 0, we selected the *M* value for which no significant increase in likelihood could be detected. Tree topology and admixture events were visualized using the internal TreeMix plotting function. In addition, interspecific admixture events identified by TreeMix were formally tested using the f_4_ statistics (*34*) implemented in the *fourpop* module of TreeMix v1.13 (*33*). Standard errors for f_4_ statistics were calculated in blocks of 500 SNPs.

### SV analysis

Patterns of genomic divergence (*F*_ST_) and outlier analysis on the RADseq data revealed that sites with elevated divergence between high- and lowland species were generally clustered into contiguous genomic regions that potentially comprise SVs like inversions or translocations. Using resequencing data, we searched for the presence of SVs in all scaffolds containing at least 10 consecutive 20-kb windows (200 kb in total) with *F*_ST_ > 0.1 in the overall comparison between the high- and lowland individuals (Fig. 4A). This selection procedure resulted in 12 scaffolds with continuous regions of elevated divergence. Each region contained at least one RADtag with an SNP identified as outlier in a minimum of two within-island ecotype comparisons, with a total of 108 outlier RADtags (57% of all identified outlier RADtags) across all 12 regions combined, which supports their association with high-lowland divergence (Fig. 4 and data S6).

We used BreakDancer v1.3.6 (*78*) to screen for anomalies in the insert size or orientation of read pairs that flanked each genomic region of elevated divergence. More precisely, BreakDancer v1.3.6 was run on all individual bam files and we searched for SVs whose breakpoints (i) are located within ±20 kb of the boundaries of the region with elevated divergence, (ii) have a maximal quality score of *Q* = 99, and (iii) are supported by a significantly different sequencing coverage between high- and lowland individuals (Welch *t* test; *P* < 0.05). Regions matching these criteria were considered as contiguous SV for further analysis.

We tested if the sequence composition at SV corresponds to the presence of distinct high- and lowland alleles by means of a PCoA on SNPs located within each SV. If haplotypes at the SV represent distinct alleles, then we expect individuals in the PCoA to be clustered into three distinct groups corresponding to individuals homozygote for the highland allele, individuals homozygote for the lowland allele, and a group of heterozygote individuals that are situated intermediate between both homozygote groups. To further confirm that the three clusters correspond to individuals with different genotypes for distinct highland or lowland alleles, we calculated average nucleotide diversity (π) at the SV and tested if π is significantly higher in the individuals in the heterozygote cluster compared to those in the two homozygote clusters. PCoA was performed with the adegenet 2.1.3 (*68*) package in R v.4.0.3 using SNPs located within each SV and filtered with VCFtools v0.1.16 (*66*) for a genotype quality of >30 and presence in all 32 resequenced individuals and kept 1 of 1000 SNPs to reduce computational time.

We tested if the SV reduced recombination between high- and lowland associated alleles by calculating pairwise *r*^2^ values between SNP genotypes across the entire length of the selected scaffolds and plotted the distribution of SNPs that are in perfect linkage disequilibrium (*r*^2^ = 1). If the SV suppresses recombination between both alleles, then *r*^2^ = 1 values are expected across the entire length of the SV. We further compared patterns of *r*^2^ between this set that includes all individuals and a set that only includes individuals that are homozygous for the allele associated with the lowland ecotype and, thus, expected to show patterns of free recombination. *r*^2^ calculations were performed with VCFtools v0.1.16 (*66*) based on the same variant call format (vcf) file as used for the PCoA (min GQ > 30, genotypes present in all individuals) but additionally filtered for a minimum allele frequency of 0.05 and an additional SNP thinning of either 1/1000 or 1/10,000 to reduce the number of SNPs to less than 500. Suppression of recombination along the SV of interest was further tested by comparing profiles of nucleotide diversity (π) between individuals that are homozygous and heterozygous for the SV, wherein heterozygous individuals are expected to consistently show higher nucleotide diversity compared to homozygotes across the entire length of the SV.

Phylogenetic relationships between the haplotypes located at the SV were estimated by ML with IQ-TREE (*71*), specifying 1000 ultrafast bootstrap samplings (*72*). Before tree estimation, best-fitting substitution models for each window were selected using ModelFinder (*73*) as implemented in IQ-TREE. We only included individuals that are homozygous for the SV to estimate phylogenetic relationships because phasing errors in individuals that are heterozygote for the highly divergent alleles may lead to erroneous recombinant haplotypes and highly inaccurate phylogenies (*79*).

We estimated the timing of the divergence between high- and lowland alleles across the different SVs using the divergence between the *Calosoma* species from the Galápagos and the closest extant mainland species *C. sayi* as calibration point. The timing of the split between the Galápagos species and *C. sayi* was first estimated from a time-calibrated phylogeny using sequence data of the mitochondrial genes *cox*I, *cytb*, and *nd1* obtained from 72 *C. granatense*, 13 *C. linelli*, 16 *C. leleuporum*, 11 *C. galapageium* individuals, and a single individual of the mainland species *C. macrum*, *C. marginale*, *C. sayi*, *C. scrutator*, and *C. wilcoxi* (*28*). Tree reconstruction was performed with BEAST v2.6.0 (*80*) using the substitution rate of 0.01342 subs/s per Myr per liter for mitochondrial genes in Coleoptera (*81*) and specified the TPM2 + F + R3 substitution model selected by ModelFinder implemented in IQ-TREE (*71*), a strict clock model, empirical base frequencies, and Yule tree prior. The outcome of this analysis estimated the split between the Galápagos species and *C. sayi* at 6.5 mya (95% highest posterior density: 5.33 to 7.66 mya).

Subsequently, we used this time calibration point for tree construction based on the sequences of each SV (including *C. sayi*) to obtain the estimated divergence time of the alleles selected in high- and lowlands. For each SV, we specified a clade with individuals with the highland allele and a clade with individuals with the lowland allele, which were both constrained to be monophyletic if supported (>95%) by our ML analysis. We specified the best-fitting substitution model selected by ModelFinder as implemented in IQ-TREE, a random local clock model, empirical base frequencies, and Yule tree prior.

We further used the estimated divergence time of *C. sayi* to estimate the timing of the divergence between the different species in this radiation by running a multispecies multilocus coalescence analysis with *BEAST (*80*) based on a random selection of 50 windows of 20 kb. We first selected 100 windows with a custom python script (SelectRandomWindows.py), which were then written to gff format specifying the start and end positions of each window. Fasta files containing the individual sequences for each window were then extracted with the vcf2fasta.pl tool (https://github.com/santiagosnchez/vcf2fasta), using the genome assembly, genome-wide VCF, and gff file specifying the locations of the 20-kb windows as input files. We used IQ-TREE (*71*) to infer the best-fitting substitution model for each window and randomly selected 50 windows for which ModelFinder reported an HKY substitution model as this allowed us to specify the same HKY substitution model for all windows simultaneously in the *BEAST analysis. Like our previous analysis on individual SVs, we specified a random local clock model, empirical base frequencies, and a Yule tree prior. All BEAST v2.6.0 (*80*) analyses were run for 50M generations, and we only used samples from the stationary phase of the Markov chain, comprising at least 25M generations, for consensus tree construction and divergence time estimation.
